# Phyto-Fenton remediation of a dichloro-diphenyl-trichloroethane contaminated site in Ha Tinh Province, Vietnam

**DOI:** 10.1038/s41598-022-20687-6

**Published:** 2022-09-30

**Authors:** Hieu Minh Dang, Cong Huu Vo, Yoshihiko Inagaki, Nhung Thi Dao, Trinh Dinh Tran, Thao Minh Tran, Thinh Thi Nguyen, Hang Thi Thuy Ho, Vien Duc Tran, Yutaka Sakakibara

**Affiliations:** 1 Vietnam Japan University, Vietnam National University, Hanoi, Vietnam; 2 Faculty of Natural Resources and Environment, Vietnam National University of Agriculture, Trau Quy, Gia Lam, Hanoi, Vietnam; 3 School of Creative Science and Engineering, Waseda University, Tokyo, Japan; 4 University of Science, Vietnam National University, Hanoi, Vietnam; 5 University of Technology and Education, The University of Danang, Danang, Vietnam

**Keywords:** Pollution remediation, Soil microbiology, Environmental sciences

## Abstract

A field trial was conducted at a site in Cam Binh commune, Ha Tinh province, Vietnam, highly contaminated with organo-pesticides. The phyto-Fenton process was applied to remove pesticide residues in soils. In addition to magnetite (Fe_3_O_4_) materials added to the soils, fertilizers and elicitors for oxidative burst were also added in the different experimental treatments. Dichloro-diphenyl-trichloroethane (DDT) and isomers were removed in all experimental lots. The removal efficiency was highest in lot B1, a site where only iron materials were added. The removal efficiency and the final content of DDTs in B1 were 98.4% and 0.009 mg kg^−1^, respectively. In the presence of elicitors, the conversion of DDT to dichloro-diphenyl-dichloroethylene was more favorable. Analysis of soil properties indicated that the phyto-Fenton process can occur at neutral soil pH, and when there are only small changes in soil organic carbon content and cation exchange capacities. Shifts in the composition of the microbial communities were observed. Further studies on the interactions between materials added to soil, plants, and the soil microbiome are needed to understand the mechanism of action of the phyto-Fenton process during soil remediation.

## Introduction

Soil contaminated with dichlorodiphenyltrichloroethane (DDT) isomers and its persistent metabolites dichlorodiphenyldichloroethane (DDD) and dichlorodiphenyldichloroethylene (DDE) are major concerns worldwide^1^. The transport of DDTs to surface water and sediment systems is often associated with runoff, erosion of contaminated soils^2^, or slow leaching into groundwater. DDT persists in living beings; fish and birds reportedly contain the highest levels of DDT and its metabolites in the Asia–Pacific region^3^. In Vietnam, the use of a centralised management model, inefficient storage, and extensive use of plant protection substances in the past have resulted in heavy pollution of many sites by residues of these chemicals. According to 2015 statistics from the Vietnam Ministry of Natural Resources and Environment, as of 2013 there were more than 1600 suspected contamination points of pesticide residues in water, concentrated mainly in the two central provinces of Vietnam: Ha Tinh and Nghe An^4^. The Decision 1946/QĐ-TTg signed by the Prime Minister in 2010 pledges to remediate and restore all environmental sites where plants are threatened by pollution by chemical residues^5^.

The remediation processes for DDT-contaminated sites are diverse and are based on the concentration of pollutants. Incineration is used for high concentrations of DDT. Emission at 700 °C and a transit time of 0.7 s is 6000 mg DDT + DDE/kg DDT^6^. The destruction and removal efficiency of DDT contaminated soil was 99.99% by introducing waste into a flue gas chamber at the kiln inlet of two different preheater/precalciner cement kilns^7^. Medium concentrations of DDT from residential areas have been remediated by separation and isolation of DDT. While these methods can be used to treat high concentrations and large amounts of residues, they are costly.

Previous studies have reported the use of ferrous iron (FeSO_4_) and endogenous hydrogen peroxide (H_2_O_2_) in aquatic plants to initiate the Fenton reaction for the mass generation of highly reactive hydroxyl radicals (·OH) to efficiently remove pentachlorophenol (PCP)^8,9^. The reaction is expressed in Eq. (1):

1 $${\text{Fe}}\left( {{\text{II}}} \right) + {\text{H}}_{{2}} {\text{O}}_{{{2} }} \to {\text{Fe}}\left( {{\text{III}}} \right) + {\text{HO}}^{ - } +^{\cdot} {\text{OH}}$$

When endogenous H_2_O_2_ is sufficient, ferric iron [Fe(III)] generated in Eq. (1), reacts with H_2_O_2_ to produce ferrous iron [Fe(II)] and ·OOH radicals, as shown in Eq. (2):

2 $${\text{Fe}}\left( {{\text{III}}} \right) + {\text{H}}_{{2}} {\text{O}}_{{2}} \to {\text{Fe}}\left( {{\text{II}}} \right) + {}^{ \cdot }{\text{OOH}} + {\text{H}}^{ + }$$

The use of Fe(III) to generate highly oxidative species is commonly defined as a Fenton-like reaction^10^. The utilization of H_2_O_2_ from plants for Fenton-like reaction has further elaborated in a so-called “phyto-Fenton technique”^8^. This technique overcomes the drawbacks of conventional Fenton techniques in that it does not require a continuous supply of either H_2_O_2_ or iron materials. The phyto-Fenton is potentially a low-cost and high-performance technique for the phytoremediation of persistent organic pollutants.

Nanosized iron particles are beneficial for the transformation and detoxification of organochlorine and polychlorinated pesticides. However, the fate and ecosystem persistence of metal oxide nanoparticles are concerning^11^. We conducted a pilot-scale application of the phyto-Fenton process to remove DDT in a contaminated site in Yen Dung district, Bac Giang province, Vietnam. The presence of vetiver and added nano-Fe_3_O_4_ increased the removal rate of DDTs^12^. However, the effects of bacterial communities and oxidative bursts on the removal of DDTs from contaminated soils were not elucidated.

Chitin is a natural polysaccharide found in the outer shells of crustaceans, such as crab and shrimp. Chitosan is a biopolymer that is obtained from chitin under strongly alkaline conditions or is formed by enzymatic deacetylation of chitin. Chitooligosaccharides, which have higher water solubility than chitin or chitosan, are generated by depolymerisation of chitin or chitosan. Chitooligosaccharides have < 20% degree of polymerisation and an average molecular weight < 3900 Da^13^. These compounds are extremely biocompatible and biodegradable, which makes them suitable for a broad range of applications, from drug delivery to food additives^14^. Since amino and hydroxyl functional groups on the polymer chains serve as binding sites to form complexes with heavy metals, chitosan and/or its derivatives as soil amendments can enhance phytoremediation for the removal of heavy metals from contaminated soils^15–17^. Chitosan is also used for water purification as a flocculent and coagulant, and for wastewater treatment as an adsorbent to remove contaminants, such as heavy metals, dyes, pesticides, and antibiotics^14^.

In agriculture, chitosan and its oligomers have been applied as potential biostimulants and elicitors to induce plant defence mechanisms to mitigate the adverse effects of biotic stresses by bacteria, fungi, and nematodes, as well as abiotic stresses that include water deficit, salinity, heat stress, and heavy metal toxicity, leading to the synthesis of reactive oxygen species (ROS)^18^. Wang et al*.*^19^ observed H_2_O_2_ accumulation in a tobacco cell suspension culture induced by oligo-chitosan. However, excess ROS can damage proteins, lipids, and nucleic acids, which are highly toxic^20^. To control ROS production and scavenging, plants have developed antioxidative systems using enzymes and antioxidants^21^. Reportedly, chitosan treatment enhances antioxidant enzyme activity via H_2_O_2_ signalling pathways^18^.

This article reports a field trial conducted at a highly DDT contaminated site in Ha Tinh province, Vietnam. In the trial, the use of chitin- and chitosan-oligosaccharides was anticipated to further facilitate the production of ROS by plants, particularly H_2_O_2_ and ^**·**^OH, thus leading to the enhancement of the performance of the phyto-Fenton process. This work aimed to evaluate and compare the degradation of DDTs and lindane in contaminated soils by the phyto-Fenton process via (1) a magnetite catalyst/vetiver system, and (2) a combination of magnetite with oxidative bursts using chitin- and chitosan-oligosaccharides (i.e. NA-COS and COS-Y)/vetiver systems as elicitors^22^.

## Results and discussion

### Soil content of DDTs at the trial site

Soil samples from the trial site and two other locations in Vietnam that were collected in a preliminary survey in 2019 were analysed for the contents of DDTs and lindane. As shown in Table 1, high levels of DDTs were present in all samples, that were many times higher than the permissible threshold of 0.01 mg kg^−1^ for DDTs in soil set by the national technical regulation (QCVN 15:2008/BTNMT)^23^. In the Cam Binh commune, where the trial took place, the content of DDTs ranged from 74.3 to 148.9 μg kg^−1^ dry soil, which was 7–15 times higher than the national standard threshold. Lindane appeared at a very trace level and was markedly below the national standard threshold of 0.01 mg kg^−1^ for lindane in soil. In particular, in this survey, all samples were taken at or near an old plant protection chemical storehouse in Nam Dinh province, which had very high concentrations of DDTs and lindane, exceeding the standard threshold by 10 times. The sample taken in Doi Lim, Bac Ninh province, showed a similar level of contamination as the samples at Cam Binh commune. In the present trial, samples at the site were collected at the beginning of the trial in December 2020. High concentrations of DDT and its metabolites were detected; the levels ranged from few to tens of times higher than the national standard threshold. In particular, the DDT content in one sample was 1090 μg kg^−1^, 109 times higher than the national threshold and approximately ten times higher than the highest DDT content detected in soil samples from Bac Giang province, where another Phyto-Fenton trial was conducted in 2017^12^. This is the reason why the present field trial was conducted in Cam Binh commune, Ha Tinh province.

**Table 1:** Contents of organochlorine pesticides in contaminated soils collected from three different locations in Vietnam.

Contaminant (μg kg^−1^)	Sample					
	CB01	CB05	ND15	ND801	DL	CB
DDT	62.27	128.9	301.54	889.83	68.90	49–1090
DDD	2.76	4.38	47.69	20.92	2.74	0–92
DDE	9.26	15.66	15.43	59.32	10.82	0–23
Lindane	nd	1.11	323.20	95.41	nd	nd

*nd* indicates not detectable.

CB01 and CB05 are samples taken in Cam Binh commune, Cam Xuyen district, Ha Tinh province. CB01 was sampled right at the place where stood the old storehouse and where the field trial took place. CB05 was sampled in the surrounding area at 5 m from CB01. ND15 and ND801 were sampled at a 15 m distance nearby the storehouse and right at the old storehouse for plant protection chemicals in Nam Dinh province, respectively. DL was sampled at a contaminated area in Doi Lim, Bac Ninh province. CB01, CB05, ND15, ND801, and DL were taken in a survey in 2019, while CB, named for samples taken at the trial site, were obtained at the starting time of the field trial in December 2020.

### Distribution of DDTs in soil at the trial site

**Figure 1:**
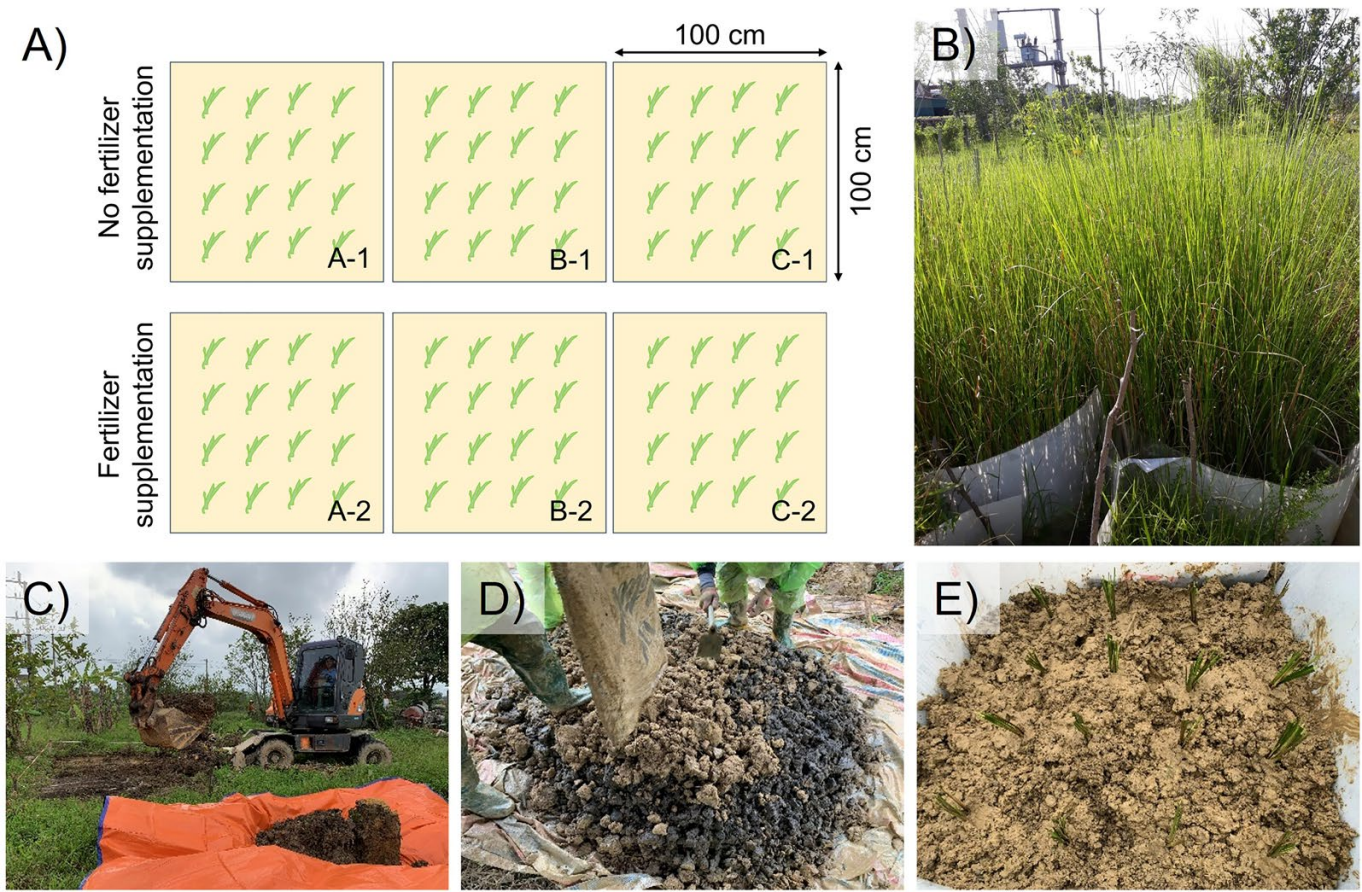
The field trial design. (**A**) Experimental layout; (**B**) selected fresh vetiver before planting; (**C**) soil excavation; (**D**) soil mixing with iron oxide nanomaterials; and (**E**) an experimental lot with vetiver planted.

The initial concentrations of DDTs were measured in six experimental lots (A1, A2, B1, B2, C1, and C2), as shown in the experimental layout in Fig. 1A. The percentage of DDT and its metabolites in each lot is shown in Supplementary Fig. 1. The DDD and DDE metabolites of DDT were detected only in lots C1 and A1. The limit of detection (LOD) of our analytical procedure was 0.005 mg kg^−1^ for the organochlorine pesticides (OCPs). According to the World Health Organisation, technical DDTs are a mixture of approximately 77% p,p′-DDT, 15% o,p′-DDT, and trace amounts of DDE and DDD^24^. The United States Environmental Protection Agency estimated that the half-life of DDTs can reach 15 years in soil^25^. In the environment, DDT undergoes slow degradation to DDE and DDD, which are more stable and persistent than the parent compound^12^. The mechanism underlying the natural conversion of DDT can be explained by the activities of both soil microorganisms and indigenous plants^26^.

### Removal of DDTs by phyto-Fenton process

**Figure 2:**
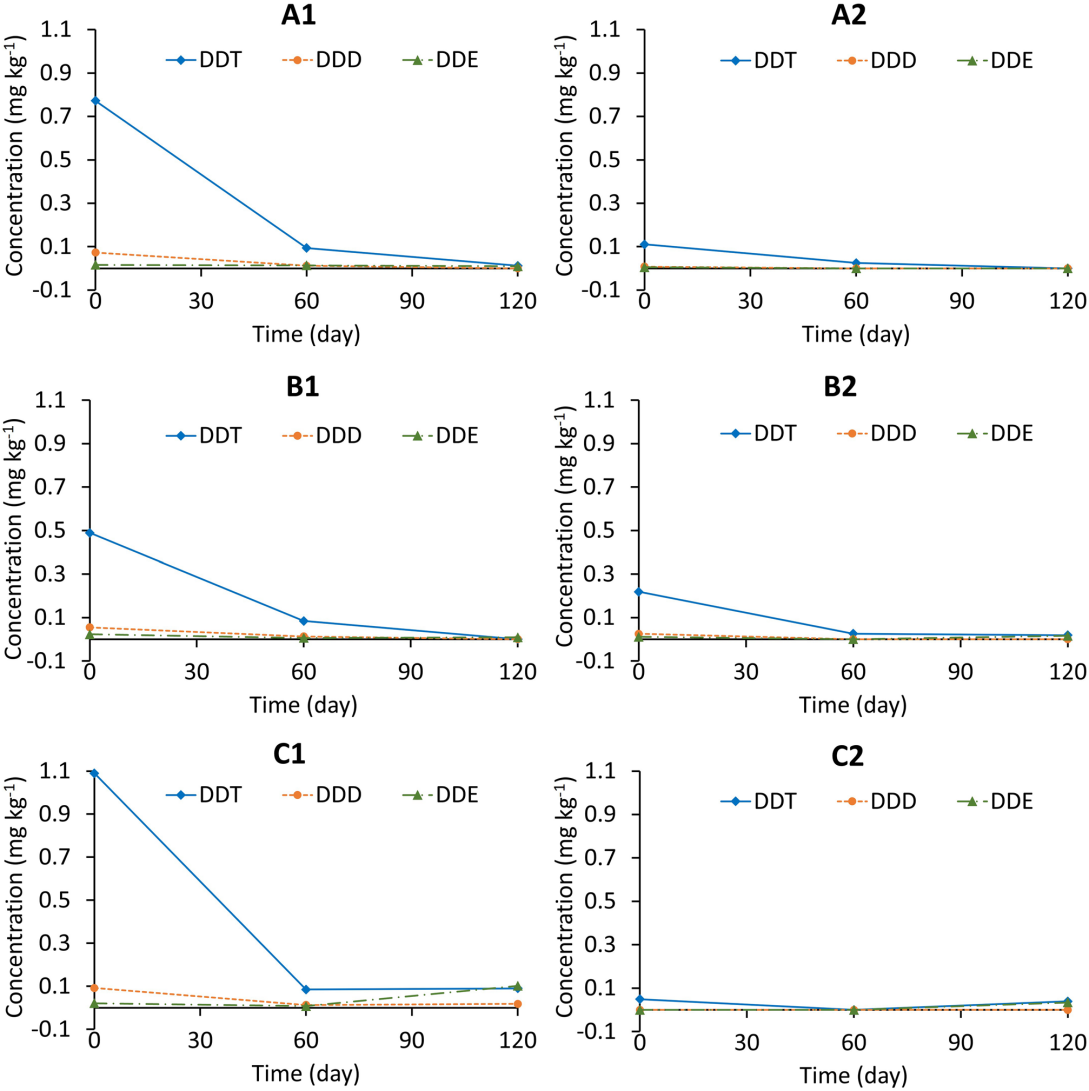
Removal of DDTs in soils at the trial site. A1, A2, B1, B2, C1 and C2 denote lots with different soil treatments as described in Table 2. Data in day 120 for A2 are missing because the concentration was below LOD.

Figure 2 shows the removal of DDTs in the six experimental lots with different treatments, as described in Table 2. As indicated by previous data on the distribution of DDTs in soil, DDT occupied the largest portion of the total DDTs in all samples at the start of the field trial. The portions of DDT showed great reductions in samples taken from all lots at 60 days after trial began, and the reduction rates were significantly slower in the subsequent 60 days. After 120 days of the trial, samples taken from lots A2 and B1 were undetectable for DDT and DDD, indicating that the content of DDTs was below the LOD of 0.005 mg kg^−1^ of the detection method. The results suggested that the phyto-Fenton process might have proceeded in all experimental lots because the soils originally already contained significantly high concentrations of Fe (approximately 200,000 mg kg^−1^). The DDE contents in all samples were most stable and even increased in lots B2 and C1 on day 120. Of note, DDE is removed slowly, but the final DDE concentration of 0.009 mg kg^−1^ was already lower than the standard threshold of 0.01 mg kg^−1^ for DDE set by the national technical regulation on the pesticide residues in the soils (QCVN 15:2008/BTNMT)^23^. The dechlorination of DDT under anaerobic and aerobic conditions leads to the formation of DDD and DDE, respectively^27^. DDE is a more persistent metabolite than DDT and DDD. Therefore, DDT transformation to DDD under anaerobic conditions is the first step in designing an effective decontamination process^28^. In this regard, the addition of magnetite to B1 treatment worked favourably.

**Table 2:** Treatment conditions.

Lot	No. plants	Iron oxide nanomaterial (g kg^−1^)	Oxidation burst (NA-COS-Y/COS-Y, g kg^−1^)	Fertiliser (g kg^−1^)	Amount of soil treated (kg)
A1	16	–	–	–	1600
B1	16	0.2	–	–	1600
C1	16	0.2	0.25/0.25	–	1600
A2	16	–	–	0.0625	1600
B2	16	0.2	–	0.0625	1600
C2	16	0.2	0.25/0.25	0.0625	1600

– indicates no chemical supplementation.

The data obtained from the samples from C2 showed a different response compared to others, demonstrating that most DDTs declined from the beginning of the trial. Better removal was expected with the addition of an oxidative burst, which entails rapid and transient production of large amounts of ROS as one of the earliest observable aspects of a plant's defence strategy^29^. However, the concentrations of DDTs during the remediation period did not decline smoothly and remained in the soil with a slight accumulation of DDE. A similar tendency was observed for lot C1. According to Zhao et al*.*^21^, under elicitor treatment, such as in lots C1 and C2, plants could produce ROS, but subsequently suffer from oxidative stress, leading to changes in contaminant detoxification. This is because plants generate more ROS with enzymes, such as NADPH oxidase and apoplastic peroxidase, which weakens ROS scavenging systems. Furthermore, the produced OH radicals might have been scavenged with abundant hydroxyl and amino groups in chitosan^18^, rather than reacting effectively with DDTs. From this point of view, we believe that further studies will be needed to elucidate an adequate amount of elicitor for the stable production and utilisation of OH radicals.

**Figure 3:**
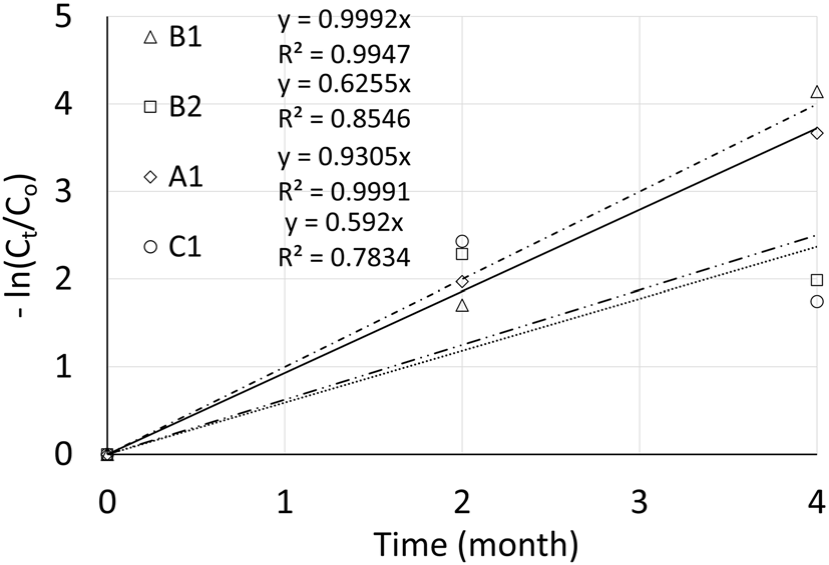
DDTs degradation kinetics. C_0_ and C_t_ denote the DDTs concentrations at the beginning of the trial and at time t (month), respectively.

A previous report on another field trial for contaminated soil remediation with the phyto-Fenton process at a site in Bac Giang province, Vietnam, indicated that the degradation of the total DDTs in the presence of nano Fe_3_O_4_ in the soil was consistent with the pseudo-first-order rate law^12^. In this study, the degradation rates of DDTs were quantitatively estimated by applying the pseudo-first-order kinetic model, as reported by Tran et al.^12^. Figure 3 shows the rate constants during degradation of total DDTs in treatments A1, B1, B2, and C1 (A1 contained iron nanomaterials in the soil). The respective constants were 0.9305, 0.9992, 0.6255, and 0.592 month^−1^. The respective coefficients were 0.9991, 0.9947, 0.8564, and 0.7834. These results imply that the degradation of DDTs in the presence of the nanomaterials in this field trial occurred according to the pseudo-first-order rate law, in agreement with the findings in the previous report. The exception was C1, where the coefficient was considerably low. Oxidation bursts with elicitors added to the soil can have various effects on the removal of DDTs from the soil by plants and the phyto-Fenton process as well as on the biodegradation potential of DDTs by soil microorganisms. The process of removing DDTs from the soil in the presence of elicitors may have to be described by a higher-order kinetic equation. It should be noted that in both treatments C1 and C2, where elicitors were present, the DDE content remarkably increased. This suggests that in these lots, the conversion of DDT to DDE was more favorable than the degradation process. From these results, a kinetic study based on stepwise degradations of DDTs should be needed to obtain more precise degradation constants and parameters.

The rate constant in treatment B1 was the highest, followed by those in treatments A1, B2, and C1. It is expected that the degradation of DDTs in soil could be enhanced in the presence of iron oxide nanomaterials. However, when fertiliser is added to the soil, it may affect or change the manner of DDTs degradation. This could be caused by induced changes in soil properties and/or the activities of above- and below-ground ecosystems, all of which can affect the degradation efficiency of organic compounds in the soil.

### Soil conditions during the Phyto-Fenton process for removal of DDTs in soil

#### Changes in soil properties

The conventional Fenton process displays its maximum ^**·**^OH production and subsequent pollutant oxidation activity in an acidic pH in the range of 2.8–3.5^30,31^. This pH medium, resulting in the use of strong sulphuric acid with ferrous and ferric catalysts and hydrogen peroxide, led to a degraded soil and microorganism community after treatment. This study evaluated pH, organic carbon, cation exchange capacity, nitrogen, and phosphorus at the initial time and after 120 days of treatment (Table 3). The observed pH was in the range of 7.54–7.84 and 7.97–8.08 at the initial time and after 120 days, respectively. In this study, magnetite (Fe_3_O_4_) which contains both Fe(II) and Fe(III) was used. Therefore, Fenton and Fenton-like reactions, as expressed in Eqs. (1) and (2) could occur. This suggests that using magnetite could overcome the drawbacks of heavy consumption of Fe(II) and generation of Fe(III) residues in the soil at neutral pH. A previous study confirmed that the phyto-Fenton reaction could occur at pH 7.5 ± 0.3^32^. Another study reported that phenol degradation by Fenton and Fenton-like reactions with commercial Fe(II, III) oxide occurred at pH 7.0 ± 0.3^33^.

**Table 3:** Properties of soils at different experimental lots during the trial.

Treatment	pH_KCl_		TOC (%)		CEC (Cmol/kg)		N (%)	P_2_O_5_ (%)
	Initial	Apr-21	Initial	Apr-21	Initial	Apr-21	Apr-21	Apr-21
A1	7.54	8.01	0.47 ± 0.02	0.65 ± 0.03	13.37	11.24	0.08	0.07
A2	7.76	8.08	0.54 ± 0.02	0.78 ± 0.02	14.25	12.06	0.09	0.12
B1	7.63	7.97	0.44 ± 0.03	0.70 ± 0.02	13.18	11.34	0.08	0.07
B2	7.84	8.08	0.51 ± 0.03	0.79 ± 0.01	14.35	12.84	0.09	0.09
C1	7.66	8.04	0.54 ± 0.02	0.70 ± 0.02	11.47	10.74	0.10	0.07
C2	7.69	7.98	0.60 ± 0.02	0.71 ± 0.02	12.12	12.92	0.10	0.10

The ability of chitosan and chitooligosaccharides to chelate minerals and other nutrients improves soil fertility, enhances nutrient uptake by plants, promotes plant growth, and modulates plant mineral concentrations^17,18^. Guo et al.^15^ demonstrated that the application of water-soluble chitosan significantly increased amino and hydroxyl groups in soils using Fourier transform infrared spectroscopy analysis. This increase might have enhanced the formation of iron complexes, thereby promoting absorption of iron from soils by plants. This could be the underlying mechanism explaining the observation that Fe concentrations in plots with Fe were similar to those in plots without Fe.

**Figure 4:**
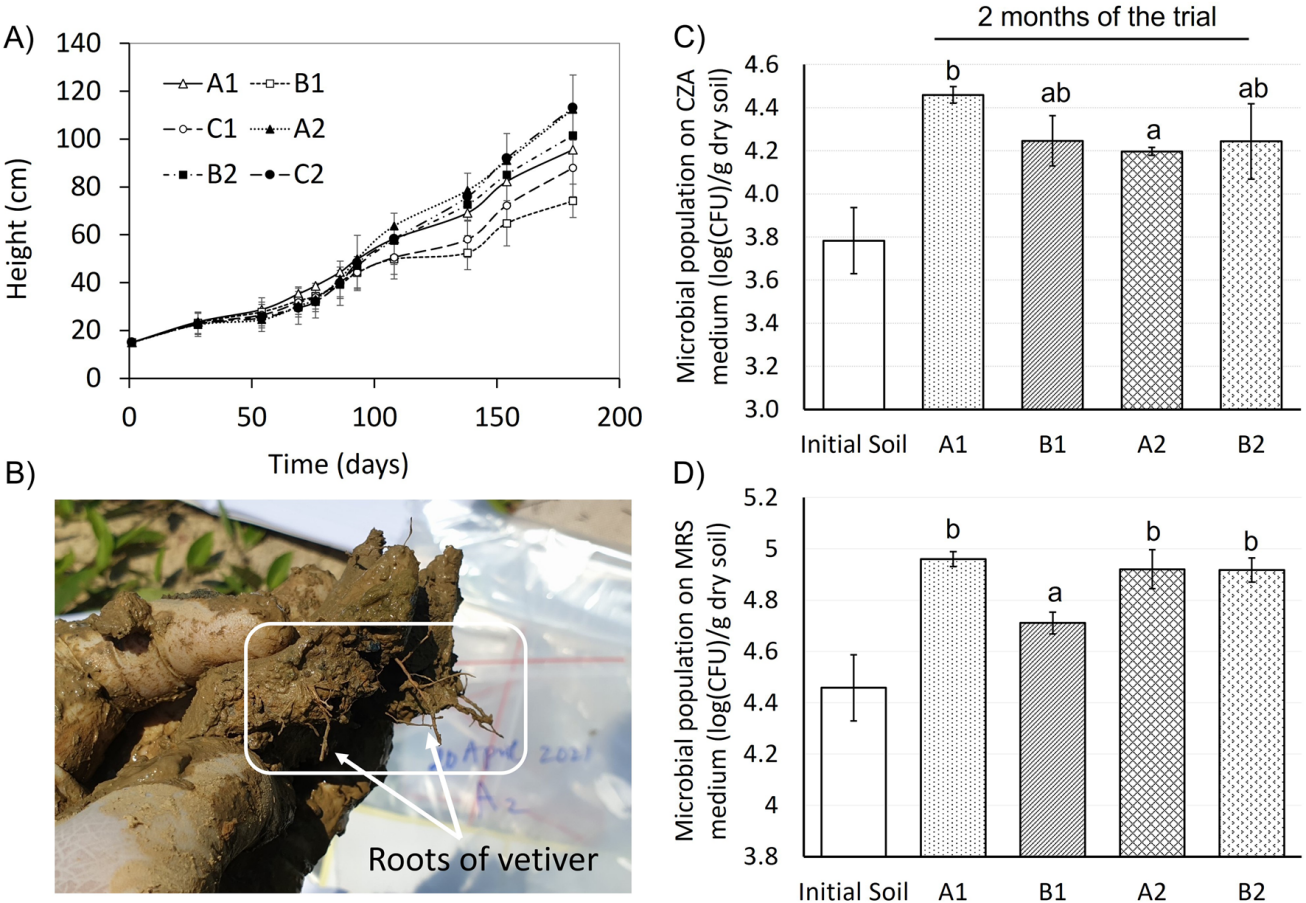
Vetiver growth during the court of experiment. (**A**) Vetiver growth during the course of experiment; (**B**) roots of vetiver distributed widely in the soil samples (taken on April 20, 2021), (**C**,**D**) microbial populations in CZA and MRS media, respectively, after 2 months of the trial. Letters a and b represent significant statistical differences (Fisher’s LSD, *p* < 0.05).

The soil total organic carbon (TOC) content in all treatments increased significantly after 120 days of treatment. TOC in lots A1, B1, and C1 increased from 0.44–0.54% to 0.65–0.70%, whereas those in A2, B2, and C2 raised from 0.51–0.60% to 0.71–0.79%. The increase in soil organic carbon was associated with the growth of vetiver. Figure 4A shows the heights of vetivers in different treatments. The heights in A2, B2, and C2 were the highest 120 days after planting. Interestingly, the root system expanded widely in soil samples (Fig. 4B), which could contribute to the formation of large amounts of ^**·**^OH by a phyto-Fenton process when roots approach magnetite in soil. On the other hand, the cation exchange capacity (CEC) in soils of all treatments showed only slight changes after 120 days. Considering the remediation of a contaminated site, which is mainly composed of concrete and clay, the soil organic carbon should meet the topsoil for agriculture, which is approximately 7 g kg^−1^^34^.

#### Changes in soil microbial community

Soil microbial communities are integral components of terrestrial ecosystems and comprise both eukaryotes (fungi, yeast, and protists) and prokaryotes (archaea and bacteria). Ecosystem functioning depends on the diversity and activity of the below-ground microbial system. In a field trial conducted in Ha Tinh province, soil microbial communities were affected by abiotic factors including temperature, humidity, soil texture and composition, and chemicals in the soil (e.g. Fe_3_O_4_), and biotic factors, such as interactions between microorganisms and microorganism and plants (vetiver). In a small trial area, since soil texture and climate conditions would be the same at all experimental slots; the influential factors would be the presence of plants, iron materials, supplemented fertiliser, and added elicitors. The compositional shifts in the microbial communities of Ha Tinh soil samples were assessed by quantifying the changes in the microbial populations on Czapek–Dox agar (CZA) and De Man, Rogosa and Sharpe (MRS) media.

The microbial populations increased in all experimental lots after 2 months of the trial. On CZA medium, the microbial population in lot A1 was the highest and was significantly different from that in lot A2 and relatively different from that in lots B1 and B2 (Fig. 4C). The CZA populations of soil supplemented with either fertiliser (A2), Fe materials (B1), or both fertiliser and Fe materials (B2), did not show significant differences after 2 months of the trial. On MRS medium, the microbial population in soil from lot B1 at 2 months was significantly lower than that in the other lots (Fig. 4D). This indicates that different materials supplemented to the soil could affect different soil microbial communities. Although there have been few studies on the effects of iron oxide nanomaterials on the soil microbiome in the soil environment, some data suggest that nanosized ZVI (nZVI) does not affect the microbial population^35^. However, iron oxides can shift the soil microbial community composition in iron-abundant soils to increase the tolerance for substrate deficiencies^36^. Studies with other metal nanoparticles have shown an influence on the structure and function of the soil microbiota^37–39^. In the present trial, the microbial populations in lot B1, where the soil was supplemented with only Fe, were significantly lower than those in the other lots. The removal efficiency of DDTs in lot B1 was the most favourable. This suggests that the phyto-Fenton process could be the key driver in the removal of DDTs from this lot. Further studies on the interactions between the soil microbiome and iron oxide materials under the phyto-Fenton reaction are needed to elucidate the underlying mechanism of action during soil remediation by the phyto-Fenton process.

#### Removal efficiency

The removal efficiencies of DDTs at contaminated site in Ha Tinh reached 82.5–98.4% (Table 4). The removal efficiency in lot B1 was the highest at 98.4% after 120 days of the trial, followed by the efficiencies in lots A1 and B2 (97.4 and 86.3%, respectively). The final content of detached DDTs was 0.009 mg kg^−1^ in B1, which was lower than the national technical threshold for DDT in soil^23^. This is because both DDT and its metabolites, DDD and DDE, were efficiently removed, whereas DDD and DDE in the other lots showed slow degradation and remained in significant amounts after 120 days of the trial. In the case of treatment A2, the initial content of DDTs was low compared to the other lots. The final concentration of DDTs in A2 was undetectable using the current analytical method. In addition, the removal efficiencies in all lots, except C1 and C2, in the present field trial were much higher than those reported in a trial conducted at a contaminated site in Bac Giang province, with an efficiency of 71.8% and a final concentration of DDTs of 0.0208 mg kg^−1^ after 140 days of phyto-Fenton remediation^12^. The level was still twice the national technical threshold for DDT in soil. Supplementation of oxidative burst produced lower removal efficiencies; the final concentration of DDTs was approximately one order of magnitude higher than that of the phyto-Fenton process. This again suggests that NA-COS and COS-Y chitin- and chitosan-oligosaccharides, respectively, might not work as effectively as expected for the elicitors for oxidative burst in vetivers. Therefore, further studies on the application of oxidative bursts and effective elicitors should be conducted^30^.

**Table 4:** Removal efficiencies after 120 days of the field trial.

Treatment	Ha Tinh trial (2021)		Bac Giang trial (2017)	
	RE (%)	Final DDTs (mg kg^−1^)	RE (%)	Final DDTs (mg kg^−1^)
A1	97.4	0.022		
B1	98.4	0.009*	71.8	0.0208**
C1	82.5	0.210		
A2	~ 100%***	nd		
B2	86.3	0.035		
C2	–	–		

*The concentration is below the permissible threshold of 0.01 mg kg^−1^ set by the national technical regulation QCVN 15:2008/BTNMT. **Results obtained in the previous trial^12^*.* ***Assumed value, since the final content of DDTs is undetectable or below the detection limit of the method in samples taken from this lot.

## Conclusion

This study demonstrates the efficient removal of DDTs by the phyto-Fenton process in a field trial conducted in Cam Binh commune, Cam Xuyen ward, Ha Tinh province, Vietnam, from December 2020 to April 2021. Different treatments with the supplementation of magnetite materials, fertiliser, and elicitors in soil coupled with planted vetivers were performed in the trial. The original content of DDTs in soil at the trial site ranged from 0.049 to 1.205 mg kg^−1^, which ranged from few to tens of times higher than the national permissible threshold of 0.01 mg kg^−1^ for DDT in soil set by the national technical regulation for pesticide residues in the soils QCVN 15:2008/BTNMT^24^. After the trial, DDTs in all experimental lots were efficiently removed. The removal efficiency was the highest in lot B1, where only magnetite was added to the soil. The efficiency and final content of DDTs was 98.4% and 0.009 mg kg^−1^, respectively. All treatments, except C1 and C2, had much higher DDT removal efficiencies than those achieved in a previously reported phyto-Fenton trial conducted in Bac Giang Province in 2017^12^. Changes in soil properties in the presence of different materials in the soil during the present trial were also assessed. Soil properties indicated small changes in the soil organic carbon content and cation exchange capacities. Examination of soil microorganisms indicated shifts in the composition of soil microbial communities. More studies on the interactions and relationships between the soil microbiome, soil structure, and materials supplemented to the soil, as well as the soil microbiome with plants are needed to further understand the underlying mechanism of action of organopesticide removal by the phyto-Fenton process.

## Methods

### Materials

The OCP standard (2000 µg mL^−1^), containing 16 constituents, including p,p′-DDE, p,p′-DDD, p,p′-DDT, and 1,2,3,4,5-pentachloro-6-(2,3,4,5,6-pentachlorophenyl) benzene (PCB-209) internal standard, was purchased from AccuStandard (USA). Other chemicals and solvents including acetone, n-hexane, sodium chloride, anhydrous sodium sulfate, sulphuric acid, hydrochloric acid, Florisil, and activated copper were purchased from Sigma-Aldrich (Germany) and Merck (USA), and were used without additional purification. The iron oxide material (Fe_3_O_4_) used in the trial was commercial magnetite powder (CAS 1317-61-9, Sigma-Aldrich, Germany). Vetiver plants (*Vetiveria zizanioides* L*.*) were provided by the Vietnam Vetiver Network and were pre-cultivated in a field at the Vietnam National University of Agriculture. In this study, the use of plants was approved by local authorities on the principle of adherence to all local, national and/or international guidelines and legislation. The plants were freshly harvested and transferred to the trial site a few hours prior to transplantation. FJ30-10-10 inorganic fertiliser (total nitrogen 30%, P_2_O_5_ 10%, and K_2_O 10%; Fuji Bio Co. Ltd., Vietnam) was used. Chitin-oligosaccharides (NA-COS-Y) and chitosan-oligosaccharides (COS-Y) were purchased from Yaizu Suisankagaku Industry Co. Ltd., Shizuoka, Japan. These products were used as elicitors for oxidative burst in plants.

### Field trial design

#### Trial location

The trial was conducted from December 2020 to December 2021 on the remaining soil of an old storehouse in Cam Binh town, Cam Xuyen district, Ha Tinh province, Vietnam (18° 17′ 29.5″ N; 105° 57′ 31.5″ E). The site was listed as high priority for contaminant removal in a government decision signed by the prime minister in 2010 (Decision 1946/QĐ-TTg)^5^. The decision stated that the DDTs level at the site was 13–50 mg kg^−1^ of soil, which is 132–330 times higher than the permissible threshold level set by the national technical regulation. The site is also among the 160 highly persistent organic pollutant contaminated sites in Ha Tinh province, as listed in a 2015 report by the Vietnam Environmental Administrative^4^.

#### Setup at the site

To set up the experiments, the waste and remains of the storehouse’s floor were removed with a small excavator. A layer of 100 cm from the soil surface was removed. Polypropylene sheets were placed into the newly excavated soil to create 100 × 100 cm^2^ lots under different test conditions (Fig. 1A). There were two test zones, one with fertiliser supplementation at 100 g m^−2^ and the other without fertiliser. Each test zone contained three lots with different soil conditions. The excavated soil was then crushed and mixed with different materials to create different soil conditions before being poured into designated lots. The soil conditions of each experimental lot are listed in Table 2.

Figure 1B–E present images of the vetiver plants selected for uniform size, and the experimental plots prepared by digging and mixing, followed by the planting of vetivers. The entire trial area was covered with plastic sheets to prevent weeds and leakage of iron material during heavy rains. A steel fence was planted around the trial area to protect the area from the cattle and poultry from surrounding households. The trial was conducted over four consecutive months (120 days) from December 2020 to April 2021.

The above setup was designed referring to the 2017 Bac Giang trial^12^. For the sake of comparison, some details of the Bac Giang trial design are briefly described as follows: a soil surface layer of 80 cm thickness was excavated by an excavator; plastic boards were installed to create lots of 60 × 60 cm^2^ for different experimental conditions; magnetite iron nanomaterials were mixed with soil from the excavated part to different concentrations before being poured into respective experimental lots; neither fertilizer nor chitin- and chitosan-oligosaccharides were used in the Bac Giang trial; the trial was conducted in six consecutive months (August 2017–February 2018).

### Soil sample collection and analysis

#### Sample collection

Soil samples of approximately 500 g each were taken every 2 months starting from December 2020. Samples were collected from a basic mixture of five points in each plot. After sampling at the site, the samples were transported directly to the laboratory and dried naturally at 24 °C for two consecutive days in a shadowed place to avoid any further degradation of organic compounds due to direct sunlight. After removing small stones and plant debris, the samples were air-dried at 24 °C, ground, and sieved through a 0.15 mm mesh screen. The sieved soil samples were then sealed in glass bottles and stored at − 20 °C until analysis.

#### Sample preparation and analysis

The procedure for preparation and analysis of the soil samples in this study was the same as that described in a previously published report by our group when reporting another Phyto-Fenton trial for the removal of DDT contaminants from soil in Bac Giang province^12^. Briefly, the sample preparation protocol was adapted from the standard protocol of the USEPA^40^. After drying, the samples were subjected to liquid–liquid extraction of DDTs (DDT and its metabolites). Then extracts containing DDTs were concentrated to 1 mL in n-hexane solution and then analysed by gas chromatography using a Scion 456-GC apparatus equipped with a DB5 column (30 m length, 0.32 mm i.d. and 0.25 μm film thickness) and an the carrier detector (GC-ECD) using helium (99.9995%) at a flow rate of 1.5 mL min^−1^ as the carrier gas, and N2 (99.9995%) with a flow rate of 30 mL min^−1^ as the supplement gas. In addition, Fe was extracted from soils following the protocol described in ISO 14869-1:2001. The analysis of Fe in the soil samples was performed following the procedure described in USEPA Method 7000 B^40^.

The growth of vetivers on test zones was assessed via weekly measurements of plant heights (16 plants × six lots). pH_KCl_, TOC (%), CEC, nitrogen (N, %), and phosphorus (P_2_O_5,_ %) of the examined soils were measured at the beginning and 4 months after the experiment. pH_KCl_ was determined according to the ISO 10390:2005 standard. TOC, CEC, N, and P_2_O_5_ contents were determined following the national standards TCVN 9294:2012, TCVN 8568:2010, TCVN 6498:1999, and TCVN 8940:2011, respectively.

#### Microbial count

The microbial counts were conducted with a routine plate count method in the laboratory using two microbial culture media. The CZA medium contained saccharose 30 g L^−1^, sodium nitrate 2 g L^−1^, dipotassium phosphate 1 g L^−1^, magnesium sulfate 0.5 g L^−1^, potassium chloride 0.5 g L^−1^, ferrous sulfate 0.01 g L^−1^, and agarose 20 g L^−1^, final pH 7.3 ± 0.2. The other medium was MRS (Merck, Germany). CZA medium is a growth medium used to propagate fungi and other aerobic organisms in the laboratory. It is recommended for use in qualitative procedures for the cultivation of saprophytic fungi, soil bacteria, and other microorganisms. MRS medium is optimised and recommended for the isolation and growth of all species of *Lactobacilli*.

Soil samples were dried at 24 °C. One gram of each sample was scaled, well-grounded, diluted in distilled water to a certain concentration, and spread on Petri dishes containing agar medium. The dishes were kept in a microbial incubator at a temperature of 35–37 °C for 2 days. Microbial colonies that appeared were counted to calculate the microbial population per 1 g of each initial sample, expressed as colony-forming units (CFU) per gram of sample.

The collected data were imported into Excel files. Statistical analysis of the data were performed using one-way ANOVA. Fisher’s LSD tests were used to compare treatment means. All analyses were conducted using the statistical package StatPlus LE Build 7.3.0.0 for Windows (StatPlus, AnalystSoft Inc., USA). The significance level was set at p < 0.05.

## Supplementary Information

Supplementary Figure 1.

## Data Availability

The datasets generated and/or analysed during the current study are available from the corresponding author upon reasonable request.
