# Supplementary material for: Phyto-Fenton remediation of a dichloro-diphenyl-trichloroethane contaminated site in Ha Tinh Province, Vietnam
Source: Sci Rep. 2022 Sep 30;12:16460. doi: 10.1038/s41598-022-20687-6 (PMC9525602; doi:10.1038/s41598-022-20687-6)
Supplement: Supplementary file 1 — Supplementary Figure 1. [file 41598_2022_20687_MOESM1_ESM.docx]

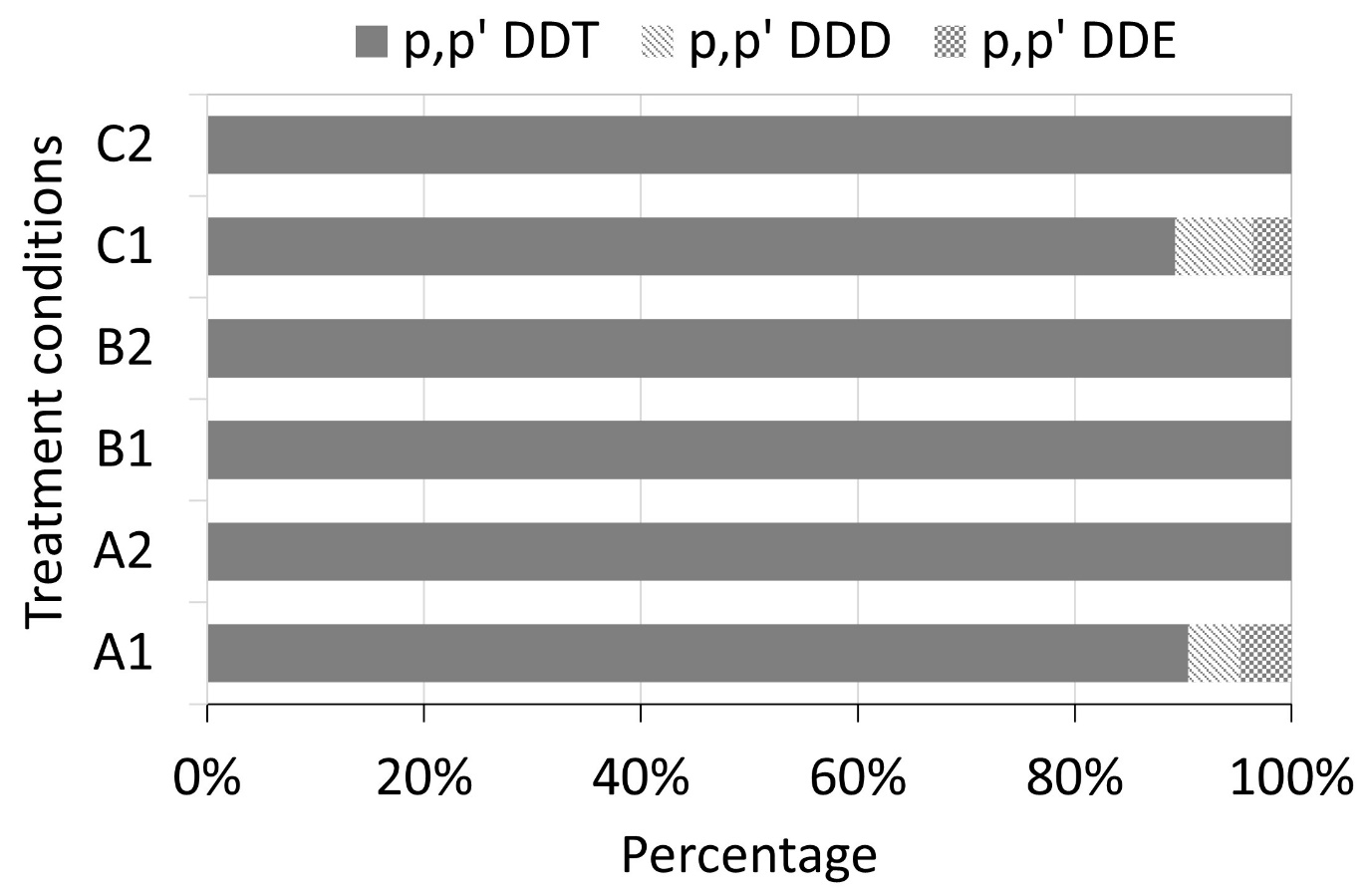


**Supplementary Figure 1.** Distribution of DDTs in examined soils in different experimental lots prior to trial implementation. A1, A2, B1, B2, C1, and C2 denote lots with different soil treatments (Table 1)*.*
